# Inhibitory effects of calcium channel blockers nisoldipine and nimodipine on ivacaftor metabolism and their underlying mechanism

**DOI:** 10.3389/fphar.2024.1403649

**Published:** 2024-09-12

**Authors:** Hailun Xia, Xinhao Xu, Jie Chen, Hualu Wu, Yuxin Shen, Xiaohai Chen, Ren-ai Xu, Wenzhi Wu

**Affiliations:** The First Affiliated Hospital of Wenzhou Medical University, Wenzhou, Zhejiang, China

**Keywords:** cystic fibrosis, ivacaftor, nisoldipine, nimodipine, drug-drug interaction, pharmacokinetics

## Abstract

Ivacaftor is the first potentiator of the cystic fibrosis transmembrane conductance regulator (CFTR) protein approved for use alone in the treatment of cystic fibrosis (CF). Ivacaftor is primarily metabolized by CYP3A4 and therefore may interact with drugs that are CYP3A4 substrates, resulting in changes in plasma exposure to ivacaftor. The study determined the levels of ivacaftor and its active metabolite M1 by ultra performance liquid chromatography tandem mass spectrometry (UPLC-MS/MS). We screened 79 drugs and 19 severely inhibited ivacaftor metabolism, particularly two cardiovascular drugs (nisoldipine and nimodipine). In rat liver microsomes (RLM) and human liver microsomes (HLM), the half-maximal inhibitory concentrations (IC_50_) of nisoldipine on ivacaftor metabolism were 6.55 μM and 9.10 μM, respectively, and the inhibitory mechanism of nisoldipine on ivacaftor metabolism was mixed inhibition; the IC_50_ of nimodipine on ivacaftor metabolism in RLM and HLM were 4.57 μM and 7.15 μM, respectively, and the inhibitory mechanism of nimodipine on ivacaftor was competitive inhibition. In pharmacokinetic experiments in rats, it was observed that both nisoldipine and nimodipine significantly altered the pharmacokinetic parameters of ivacaftor, such as AUC_(0-t)_ and CL_z/F_. However, this difference may not be clinically relevant. In conclusion, this paper presented the results of studies investigating the interaction between these drugs and ivacaftor *in vitro* and *in vivo*. The objective is to provide a rationale for the safety of ivacaftor in combination with other drugs.

## 1 Introduction

Cystic fibrosis (CF) is an inherited disease caused by a genetic mutation that affects the secretory function of the mucus glands of the respiratory and gastrointestinal systems, severely limiting the life expectancy of patients (Sanders and Fink, 2016; O’Sullivan and Freedman, 2009; Elborn, 2016). Recent studies have shown that CF is caused by mutations in the gene encoding the cystic fibrosis transmembrane conductance regulator (CFTR), and CFTR potentiators have been shown to be useful in reducing disease progression in patients with CF (Elborn, 2016; Middleton et al., 2019).

Ivacaftor is the first CFTR potentiator approved by the United States Food and Drug Administration and the European Medicines Agency for the treatment of CF alone. Ivacaftor significantly improves lung function, especially in CF patients with the G551D CFTR missense mutation (Hadida et al., 2014; Ramsey et al., 2011). Ivacaftor can alleviate the symptoms of CF patients by improving the function of CFTR on the cell surface, enhancing the transport of chloride ions, increasing negative ion conductivity, improving mucus hydration, and decreasing mucus viscosity (Yu et al., 2012; Garg et al., 2019; Harbeson et al., 2017).

With advancements in early diagnosis, newborn screening, modern medical technology, and the extensive use of CFTR modulators, the median life expectancy of people with CF in the developed world has surpassed 40 years of age (McBennett et al., 2021; Elborn, 2016). As survival time increases, the likelihood of developing other diseases, especially chronic diseases such as cardiovascular disease, gradually increases in CF patients. CF patients often require combination therapy with other medications to improve their quality of life, and this combination of medications increases the likelihood of drug-drug interactions (DDI). Ivacaftor plasma protein binding has been reported to be >97%, which may affect drug concentrations when used in combination with other drugs (Schneider et al., 2015). Although ivacaftor therapy has been well tolerated by CF patients in established clinical trials, the occurrence of adverse events has been monitored in both experimental and real-world settings (Gavioli et al., 2020; Dagenais et al., 2020). Although a safe plasma concentration range for ivacaftor has not yet been established, real-world data suggest a potential link between drug exposure and toxicity (Dagenais et al., 2020; Hong et al., 2023a; Spoletini et al., 2022).

Ivacaftor is oxidatively metabolized mainly by the CYP3A family pathway, with hydroxymethyl ivacaftor (M1) as the active metabolite (Hong et al., 2023b; Robertson et al., 2015). The FDA reported that ivacaftor monotherapy resulted in an 8.5-fold increase in the area under the plasma concentration-time curve (AUC) of ivacaftor when combined with ketoconazole, a strong CYP3A inhibitor. In combination with itraconazole, the AUC of ivacaftor increased 15.6-fold. The plasma exposures of ivacaftor are significantly increased by strong CYP3A inhibitors (Garg et al., 2019). Another study in humans found that ritonavir, a potent CYP3A4 inhibitor, increased plasma exposure to ivacaftor 7-fold (van der Meer et al., 2021). Available studies suggest that there are significant effects of potent CYP3A4 inhibitors on the plasma exposure of ivacaftor, however, the effect of other, slightly less inhibitory drugs on the metabolism of ivacaftor is unclear. Ivacaftor often needs to be used in combination with other medications to help control symptoms in people with CF, with the potential for DDI (van der Meer et al., 2021; Hong et al., 2023b; Robertson et al., 2015). Therefore, it is important to study the possibility of DDI of ivacaftor with other drugs.

To investigate the possibility of DDI between ivacaftor and other drugs, we developed and used an ultra performance liquid chromatography tandem mass spectrometry (UPLC-MS/MS) assay for this study. Firstly, enzyme incubation experiment in rat liver microsomes (RLM) was used as indicator to screen out the drugs that might have DDI with ivacaftor among 79 common drugs. The half-maximal inhibitory concentrations (IC_50_) values of 6 calcium channel blockers against ivacaftor were further investigated. Finally, we selected nisoldipine and nimodipine as drugs that might have *in vivo* inhibitory effects on ivacaftor and performed pharmacokinetic experiments in rats. In addition, the mechanism of inhibition of ivacaftor by nisoldipine and nimodipine in RLM and human liver microsomes (HLM) were investigated. We hope that the results of this study will provide a basis for the safety of ivacaftor in combination with other drugs and offer the possibility of improving the quality of life of CF patients.

## 2 Experimental

### 2.1 Materials and methods

#### 2.1.1 Chemicals and reagents

The following drugs were supplied by Beijing Sunflower Technology Development Co., Ltd. (Beijing, China): ivacaftor, fluconazole (used as internal standard, IS), and hydroxymethyl ivacaftor (M1). Nisoldipine, nimodipine, nifedipine, felodipine, lacidipine, nicardipine, and the other drugs used in our experiments were provided by Shanghai Canspec Scientific Instruments Co., Ltd. (Shanghai, China). The purity of all drugs used in the experiments were ≥98%. Details of these drugs were listed in Supplementary Table S1. Acetonitrile (HPLC grade) and methanol (HPLC grade) were supplied by Merck (Darmstadt, Germany). The Milli-Q water purification system, manufactured by Millipore in Bedford, United States, was used to prepare ultra-pure water. Reduced nicotinamide adenine dinucleotide phosphate (NADPH) was purchased from Shanghai Aladdin Biochemical Technology Co., Ltd. (Shanghai, China). RLM was prepared in our laboratory. Protein concentrations for RLM and HLM are presented in Supplementary Table S2. HLM in this experiment was provided by iPhase Pharmaceutical Services Co., Ltd. (Beijing, China). All other chemicals and biologicals in our experiments were of analytical grade or above.

#### 2.1.2 Equipment and operating conditions

The concentrations of ivacaftor and M1 were determined using ultra performance liquid chromatography tandem mass spectrometry (UPLC-MS/MS) technology. A Waters Acquity UPLC BEH C18 column (2.1 mm × 50 mm, particle size 1.7 μm) was used in the chromatographic system for separation, and the column temperature was set at 40°C. Additional conditions were set as follows: injection volume 2.0 μL, and autosampler temperature 10°C. 0.1% formic acid aqueous solution (solution A) and acetonitrile (solution B) were used as the mobile phase, and the gradient elution was as follows: 0–0.5 min at 90% A, 0.5–1.0 min at 90%–10% A, 1.0–1.4 min at 10% A, 1.4–1.5 min at 10%–90% A. The entire run time was 2.0 min, and the flow rate was maintained at 0.4 mL/min. We used a Waters Xevo TQS triple quadrupole mass spectrometer (Milford, MA, United States) with multiple reaction monitoring (MRM) in positive mode selected for the quantification of ivacaftor and M1. Monitoring the transition pairs were *m/z* 393.08→337.02 for ivacaftor, *m/z* 409.07→353.02 for M1 and *m/z* 307.10→220.00 for IS, respectively (Table 1). The collision energies of ivacaftor, M1 and IS were 11 eV, 10 eV and 20 eV, respectively.

**TABLE 1:** The quantitative ion pairs and related parameters of ivacaftor, its metabolite M1 and IS.

Compound	Parent (*m/z*)	Daughter (*m/z*)	Cone (V)	Collision (eV)
Ivacaftor	393.08	337.02	10	11
M1	409.07	353.02	20	10
IS	307.10	220.00	30	20

#### 2.1.3 RLM

Previous studies have reported the preparation of RLM, which we had modified (Wang et al., 2014). Liver was weighed and homogenized with cold 0.01 mM phosphate-buffered saline (PBS) containing 0.25 mM sucrose, the homogenate was centrifuged at 11,000 rpm for 15 min. The supernatants were then transferred to new tube and centrifuged at 11,000 rpm for 15 min. The supernatants were centrifuged at 100,000 × g for 1 h, and the pellets were resuspended with cold 0.01 mM PBS. Protein concentrations were determined by Bradford Protein Assay Kit (Thermo Scientific, Waltham, MA, United States).

### 2.2 Enzyme reaction of ivacaftor using RLM and HLM

This system was made up of 200 μL of incubation solution consisting of 0.3 mg/mL RLM or 0.4 mg/mL HLM, 1.0 mM NADPH, pH 7.4 PBS and ivacaftor. Ivacaftor was used at a range of concentrations (0.1, 1, 2, 4, 12, 16 μM) for the determination of K_m_ (Michaelis-Menten constant) in RLM. In HLM, a range of concentrations (0.1, 1, 2, 4, 12, 25 μM) of ivacaftor was used to determine K_m_. The solution should be pre-incubated at 37°C for 5 min before adding NADPH. Following the addition of NADPH, the mixture should be incubated for 40 min. The reaction should then be stopped at −80°C. After the enzyme reaction was completed, 20 μL of 500 ng/mL IS solution and 400 μL acetonitrile (protein precipitating agent) were added to the mixture. The mixture was vortexed for 2 min and then centrifuged at 13,000 rpm for 10 min. After centrifugation, 100 μL of the supernatant was quantified using UPLC-MS/MS.

### 2.3 Determination of DDI and inhibition mechanism of ivacaftor *in vitro*

The K_m_ of ivacaftor in the RLM incubation system was determined to be 8.8 μM. First, we established a culture system to screen these 79 drugs that may have an effect on the metabolism of ivacaftor. Each drug was used at a concentration of 100 μM as the inhibitor. The culture system was 200 μL and included 0.3 mg/mL RLM, 1.0 mM NADPH, PBS, ivacaftor, and inhibitor. The reaction procedure was the same as the enzyme reaction mentioned above Section 2.2.

The IC_50_ values were determined to assess the inhibitory effects of nisoldipine, lacidipine, felodipine, nimodipine, nicardipine, and nifedipine on ivacaftor in RLM. The IC_50_ values for the inhibitory effects of nisoldipine and nimodipine on ivacaftor in HLM were also determined. These drugs were dissolved and diluted using DMSO to prepare a gradient concentration of IC_50_ at 0, 0.01, 0.1, 1, 10, 25, 50, and 100 μM. The final concentration of DMSO in the culture system is less than 1%. Based on the K_m_ values in RLM and HLM, the concentrations of ivacaftor were 8.8 and 6.8 μM, respectively. The incubation and post-treatment procedures were consistent with the enzyme incubation of ivacaftor described above Section 2.2.

To investigate the type of inhibitory mechanism, we used Lineweaver-Burk plot analysis and calculation of inhibition constants (K_i_ and αK_i_), where the drug concentrations were set to be, in RLM, 2.2, 4.4, 6.6, and 8.8 μM for ivacaftor, 0, 1.64, 4.91, and 6.55 μM for nisoldipine, and 0, 2.29, 3.43, 4.57 μM for nimodipine, respectively; in HLM, 1.7, 3.4, 5.1, 6.8 μM for ivacaftor, 0, 2.28, 6.83, 9.10 μM for nisoldipine, and 0, 3.58, 5.36, 7.15 μM for nimodipine, respectively. The incubation and post-treatment procedures were consistent with the enzyme incubation of ivacaftor described above Section 2.2.

### 2.4 Study of pharmacokinetics in Sprague-Dawley rats

The animal study was supervised and approved by Ethics Committee of The First Affiliated Hospital of Wenzhou Medical University (WYYY-IACUC-AEC-2023-065). 12 Sprague-Dawley male rats (220 ± 20 g) were purchased from the First Affiliated Hospital of Wenzhou Medical University and randomly divided into three groups: group A (control), group B (nisoldipine), and group C (nimodipine), with 4 rats in each group. A 12 h fast with no restriction on water intake was conducted prior to the experiment. Ivacaftor, nisoldipine, and nimodipine were orally administered as suspensions in a 0.5% solution of sodium carboxymethyl cellulose (CMC-Na). Group B were received nisoldipine (1 mg/kg) via gavage, while group C were received nimodipine (10 mg/kg) via the same method. Group A were given the same volume of CMC-Na to serve as a control. After 30 min of inhibitor administration to rats, 10 mg/kg of ivacaftor was again administered by gavage. Tail venous blood was collected from rats at 0, 0.5, 1, 1.5, 2, 3, 4, 6, 8, 12, 24 and 48 h after ivacaftor administration. 0.3 mL of blood sample was collected and centrifuged at 8,000 rpm for 10 min at 4°C, and 100 μL of supernatants were frozen at −80°C for further processing. Before UPLC-MS/MS analysis, supernatant was mixed with 300 μL acetonitrile and 10 μL IS (500 ng/mL), and then vortexed for 2 min and centrifuged at 13,000 rpm for 10 min at 4°C. Finally, a 100 μL sample of supernatant was collected for UPLC-MS/MS analysis.

### 2.5 Data analysis

K_m_ values, IC_50_ values, Lineweaver-Burk plots and mean plasma concentration-time curves were all generated using GraphPad Prism 9.0 software (GraphPad Software, Inc., United States). Pharmacokinetic parameters of ivacaftor and M1 were obtained by analyzing them using the non-compartmental model of Drug and Statistics (DAS) (version 3.0, Mathematical Pharmacology Professional Committee of China, Shanghai, China), and a one-way ANOVA was performed to compare the pharmacokinetics of the three groups of rats using SPSS (version 26.0; SPSS Inc., Chicago, IL, United States). A *P*-value <0.05 was considered statistically significant. Data were represented as mean ± standard deviation (SD).

## 3 Results

### 3.1 Determination of ivacaftor and its metabolite by UPLC-MS/MS

**FIGURE 1:**
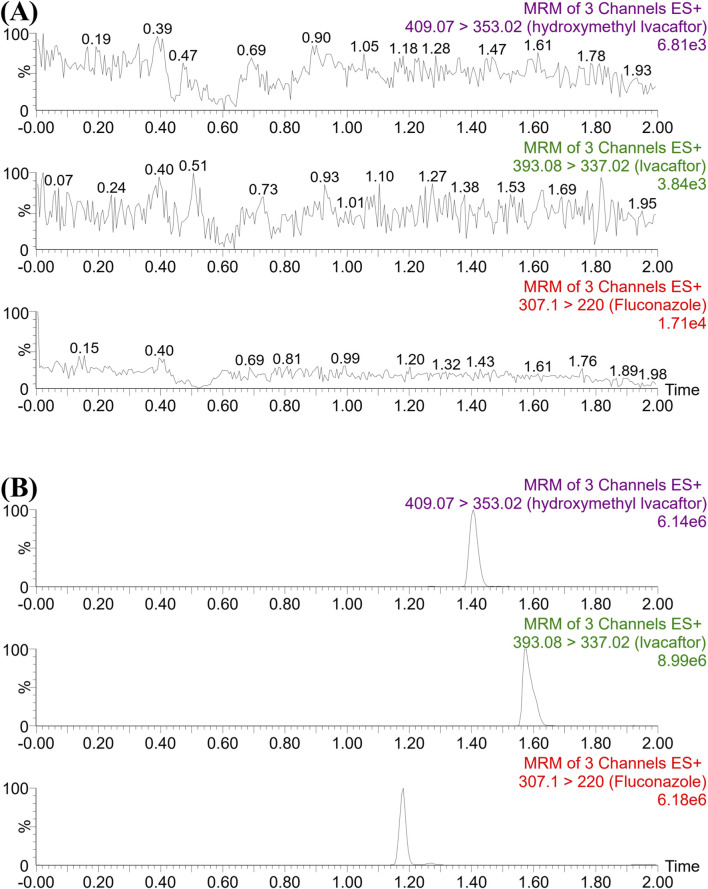
UPLC-MS/MS chromatographs of ivacaftor, M1 and fluconazole (IS). **(A)** Blank plasma sample, no analyte, no IS. **(B)** Rat plasma sample after the administration of ivacaftor.

As shown in Figure 1, the retention times of ivacaftor, M1 and IS were 1.58, 1.41 and 1.18 min, respectively. The analytes were well separated from each other, and no interfering peaks were found to affect the determination of the analytes. The standard calibration curves for ivacaftor and M1 were in the ranges of 1–1,000 ng/mL and 1–200 ng/mL, respectively, with correlation coefficients greater than 0.99.

### 3.2 Screening of drugs with inhibitory effects on ivacaftor

**FIGURE 2:**
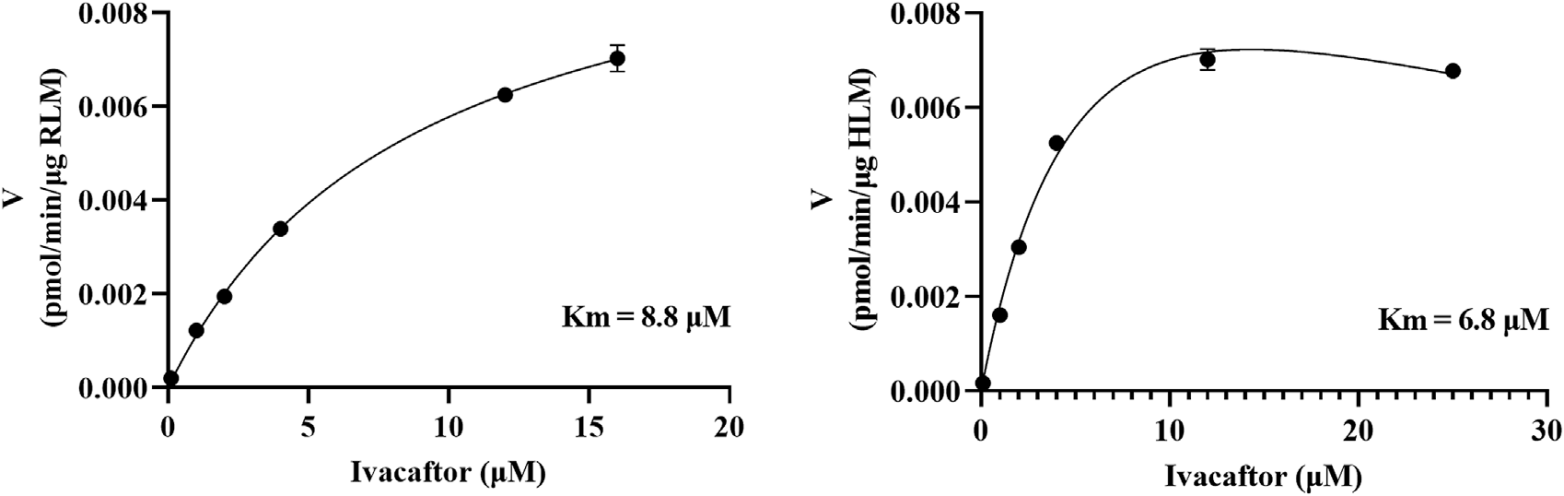
Michaelis–Menten kinetics of ivacaftor in RLM and HLM.

**FIGURE 3:**
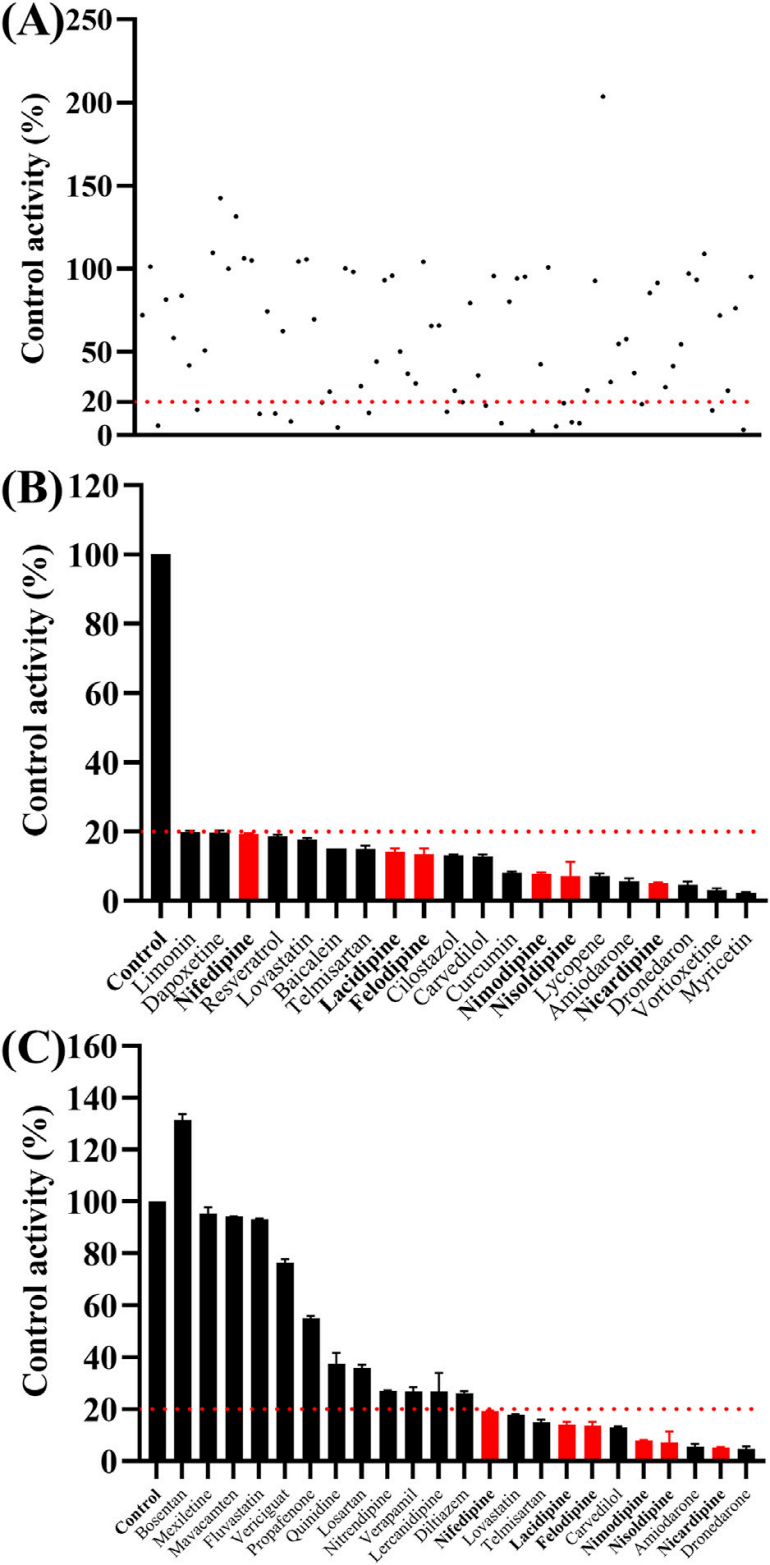
Comparison of the inhibitory effects of different drugs (100 μM) on the metabolism of ivacaftor in RLM. For all screened drugs (**(A)**, the red line represents 20%), 20 drugs with metabolic rates less than 20% of the control **(B)** and cardiovascular drugs **(C)**. Data are represented as mean ± SD.

As shown in Figure 2, the K_m_ values of ivacaftor in RLM and HLM were 8.8 μM and 6.8 μM, respectively. This experiment screened 79 potential drugs that could be combined with ivacaftor, including cardiovascular drugs, traditional Chinese medicines, and others. The results of inhibition on ivacaftor by these drugs were shown in Figure 3A. Among these, 20 drugs showed inhibition rates of more than 80% (Figure 3B). In addition, the condition of inhibition rates by 23 cardiovascular drugs were displayed in Figure 3C. We found that nicardipine, nisoldipine, nimodipine, felodipine, lacidipine, and nifedipine showed inhibitions of 94.98%, 93.06%, 92.54%, 87.18%, 86.56%, and 80.69%, respectively. These results strongly suggested a high likelihood of DDI occurring when ivacaftor is combined with a calcium channel blocker.

### 3.3 Nisoldipine and nimodipine inhibited ivacaftor metabolism in RLM and in HLM through different inhibitory mechanisms

**FIGURE 4:**
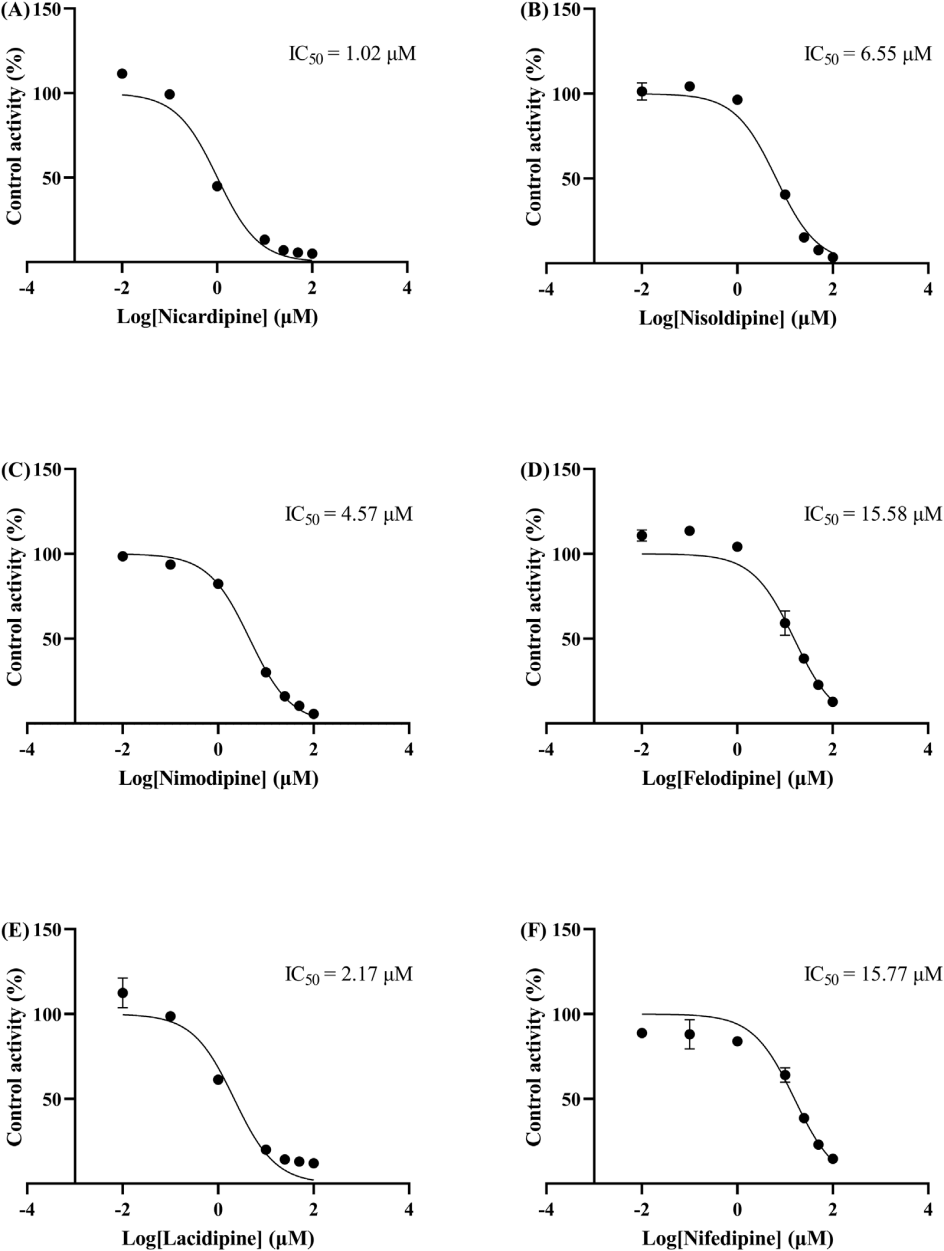
IC_50_ curves of 6 cardiovascular drugs on ivacaftor metabolism in RLM. Nicardipine **(A)**, nisoldipine **(B)**, nimodipine **(C)**, felodipine **(D)**, lacidipine **(E)**, and nifedipine **(F)**.

**FIGURE 5:**
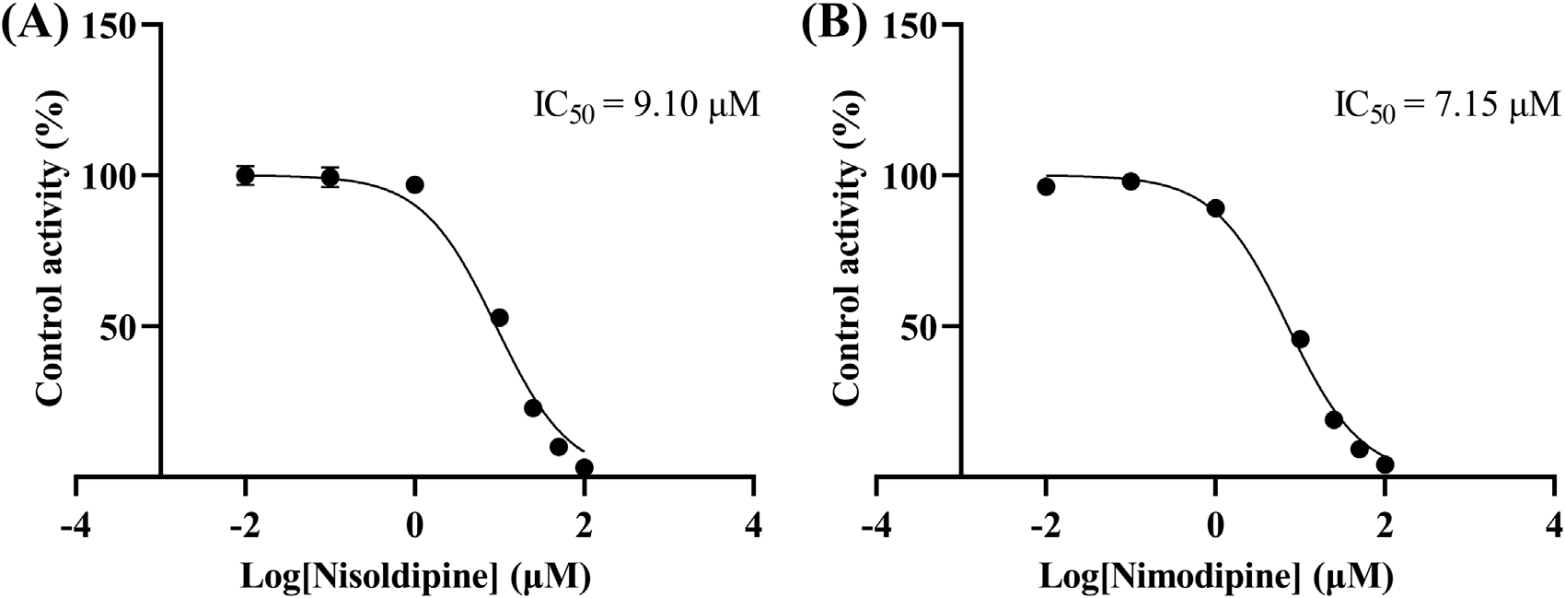
IC_50_ curves of nisoldipine **(A)** and nimodipine **(B)** on ivacaftor metabolism in HLM.

**FIGURE 6:**
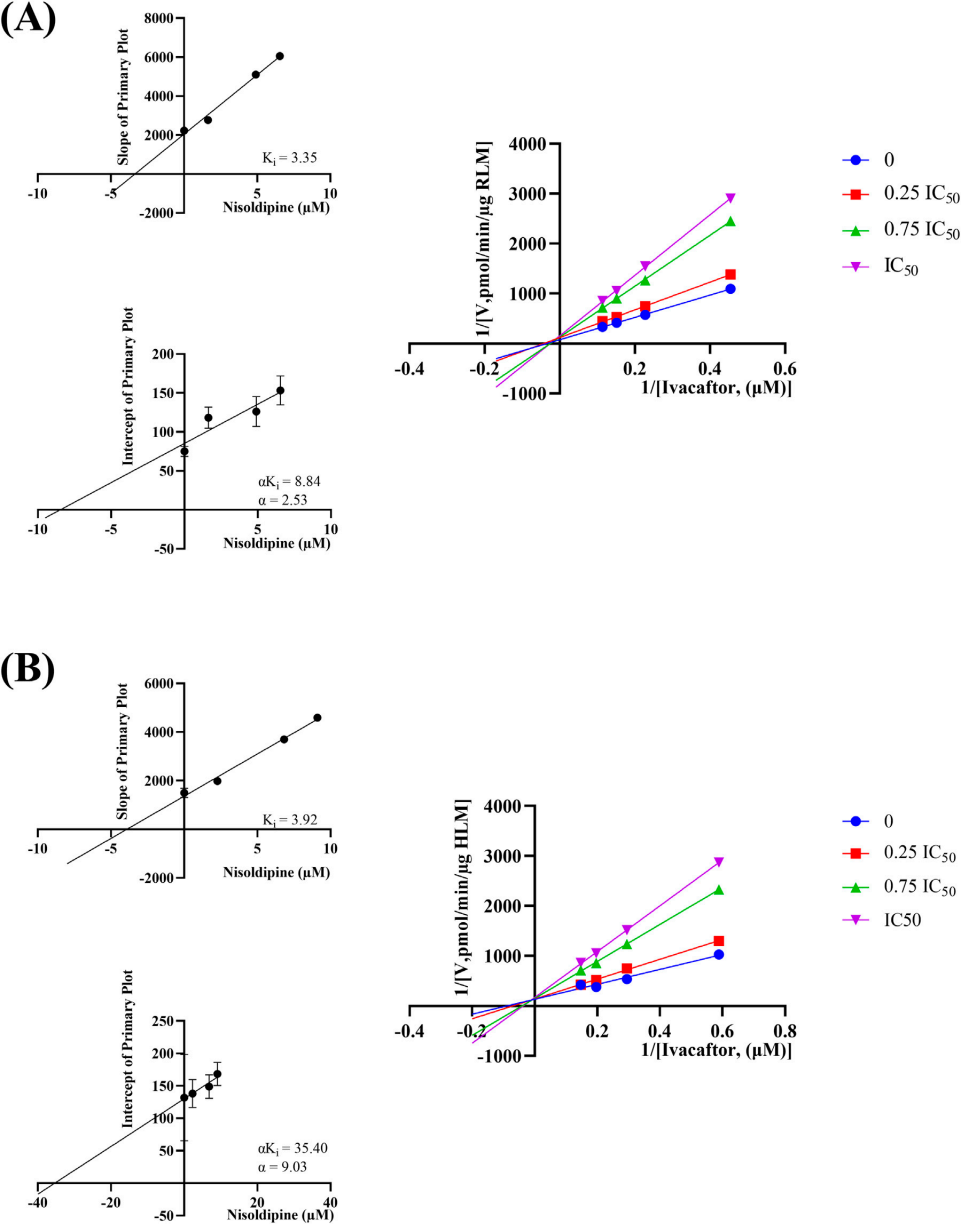
Lineweaver-burk plot, secondary diagram of K_i_ and secondary diagram of αK_i_ inhibiting ivacaftor metabolism at different concentrations of nisoldipine in RLM **(A)** and in HLM **(B)**.

**FIGURE 7:**
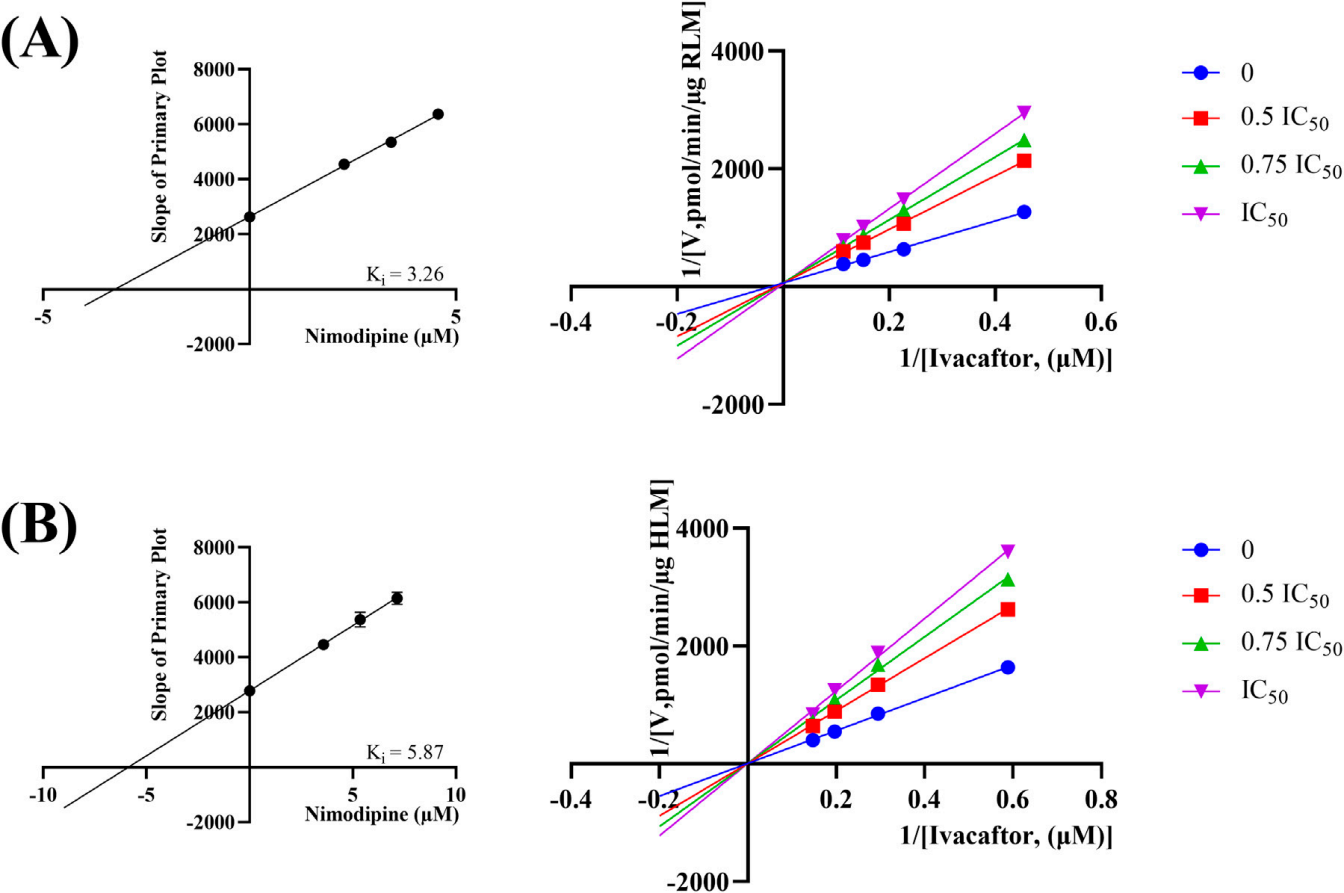
Lineweaver-burk plot, secondary diagram of K_i_ inhibiting ivacaftor metabolism at different concentrations of nimodipine in RLM **(A)** and in HLM **(B)**.

The IC_50_ curves for the metabolism of ivacaftor by the 6 calcium channel blockers in RLM were shown in Figure 4. The IC_50_ value is calculated as Y = 100/(1 + 10^^(X−LogIC50)^). The inhibition assessment indicated that M1 concentrations were significantly reduced by nicardipine, nisoldipine, nimodipine and lacidipine (with IC_50_ values of 1.02 μM, 6.55 μM, 4.57 μM and 2.17 μM, respectively). In contrast, the IC_50_ values of felodipine and nifedipine were 15.58 μM and 15.77 μM, respectively, and the inhibition of ivacaftor metabolism was weaker than that of the other 4 drugs. When the IC_50_ value <10 μM, it indicates a strong inhibitory effect on the metabolism of ivacaftor. Figure 5 showed the IC_50_ curve of nisoldipine on ivacaftor metabolism in HLM at IC_50_ value of 9.10 μM. According to Figure 6, Lineweaver-Burk plots in RLM and HLM, nisoldipine was found to inhibit the metabolism of ivacaftor through a mixed-type of non-competitive and competitive inhibition. The K_i_ values were 3.35 and 3.92, and the α values were 8.48 and 35.40, respectively (Table 2). In addition, the IC_50_ value of nimodipine in HLM was 7.15 μM. Nimodipine was found to inhibit the metabolism of ivacaftor through competitive inhibition based on Lineweaver-Burk plots in both RLM (Figure 7A) and HLM (Figure 7B). The K_i_ values were 3.26 and 5.87, respectively (Table 2).

**TABLE 2:** The IC_50_ values and inhibitory effects of nisoldipine and nimodipine on ivacaftor metabolism in RLM and HLM.

Inhibitors		IC_50_ values (μM)	Inhibition type	K_i_ (μM)	αK_i_ (μM)	α
Nisoldipine	RLM	6.55	Non-competitive inhibition and competitive inhibition	3.35	8.48	2.53
	HLM	9.10	Non-competitive inhibition and competitive inhibition	3.92	35.40	9.03
Nimodipine	RLM	4.57	Competitive inhibition	3.26		
	HLM	7.15	Competitive inhibition	5.87		

### 3.4 Effects of nisoldipine and nimodipine on the metabolism of ivacaftor *in vivo*

**FIGURE 8:**
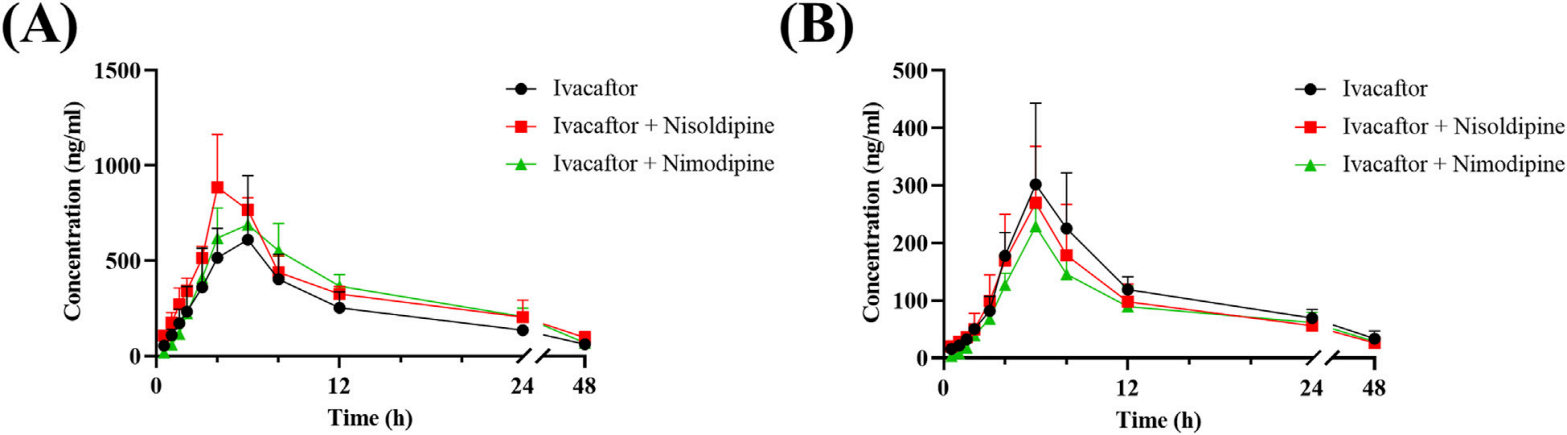
Mean plasma concentration-time curve of ivacaftor **(A)**, and M1 **(B)** in rats. Data are represented as the mean ± SD, n = 4.

The mean concentration-time curves of ivacaftor and M1 were shown in Figure 8. The main pharmacokinetic results derived from the non-compartmental modeling analyses performed on rats in the control, nisoldipine, and nimodipine groups were presented in Tables 3, 4. The results demonstrated the significant changes of pharmacokinetic parameters of ivacaftor in the presence of nisoldipine and nimodipine in rats. The administration of nisoldipine with ivacaftor significantly increased the AUC_(0-t)_ and AUC_(0-∞)_ of ivacaftor in rats, while reducing CL_z/F._ There were no significant changes in t_1/2_, T_max_ and C_max_. In detail, in rats that were given nisoldipine, the AUC_(0-t)_ of ivacaftor was increased by 0.51-fold, while CL_z/F_ was decreased by 32.11%. Therefore, it could be concluded that nisoldipine increased the exposure of ivacaftor *in vivo* after administration to rats, suggesting that nisoldipine may potentially interact with ivacaftor. Moreover, in rats given nimodipine, the AUC_(0-t)_ of ivacaftor was increased by 0.44-fold, while CL_z/F_ was decreased by 30.27%. Although there were no significant differences in the pharmacokinetic parameters of M1 (*p* > 0.05), the metabolite-parent ratio (MR, MR = AUC_M1_/AUC_Ivacaftor_) was reduced by 44.34% and 45.90% in the nisoldipine group and the nimodipine group, respectively, compared to the control group. These findings provided evidence that nisoldipine and nimodipine inhibited the metabolism of ivacaftor in rats.

**TABLE 3:** The pharmacokinetic parameters of ivacaftor in the three groups of rats (n = 4).

Parameters	Ivacaftor	Ivacaftor + nisoldipine	Ivacaftor + nimodipine
AUC_(0-t)_ (ng/mL*h)	8,408.56 ± 1,515.44	12,659.36 ± 2,103.23*	12,139.40 ± 1,270.98*
AUC_(0-∞)_ (ng/mL*h)	9,357.24 ± 1,331.88	14,042.34 ± 3,176.58*	13,297.30 ± 1,474.72
t_1/2_ (h)	12.20 ± 4.80	13.29 ± 3.93	13.03 ± 3.25
T_max_ (h)	6.50 ± 1.00	4.50 ± 1.00	5.50 ± 1.00
CL_z/F_ (L/h/kg)	1.09 ± 0.16	0.74 ± 0.15*	0.76 ± 0.09*
C_max_ (ng/mL)	675.93 ± 241.90	906.62 ± 252.57	737.89 ± 74.62

*p < 0.05, compared with the control group. Data are represented as mean ± SD.

**TABLE 4:** The pharmacokinetic parameters of M1 in the three groups of rats (n = 4).

Parameters	Ivacaftor	Ivacaftor + nisoldipine	Ivacaftor + nimodipine
AUC_(0-t)_ (ng/mL*h)	4,305.47 ± 951.85	3,606.73 ± 1,008.67	3,361.75 ± 881.14
AUC_(0-∞)_ (ng/mL*h)	4,773.01 ± 1,120.53	4,220.29 ± 1,249.82	4,258.04 ± 782.59
t_1/2_ (h)	12.68 ± 0.90	15.93 ± 3.56	22.40 ± 5.69
T_max_ (h)	6.50 ± 1.00	6.50 ± 1.00	6.50 ± 1.00
CL_z/F_ (L/h/kg)	2.17 ± 0.44	2.59 ± 1.00	2.41 ± 0.46
C_max_ (ng/mL)	317.55 ± 114.54	276.58 ± 99.87	230.37 ± 32.11

Data are represented as mean ± SD.

## 4 Discussion

CF is a genetic ailment whereby mucus obstructs the respiratory and digestive systems, necessitating long-term medication for managing its advancement. This severely compromises CF patients’ quality of life and amplifies their financial burden. Unfortunately, CF has no known cure, and patients can only retrench the disease’s progression with medication. Ivacaftor is a CFTR potentiator that can be used individually to treat CF patients with the G551D-CFTR missense mutation. As the average life expectancy of CF patients increases, the likelihood that CF patients will have chronic diseases increases, increasing the need for combination therapy with other drugs. It has been clinically reported that patients with CF may develop systemic arterial hypertension during the first week of elexacaftor/tezacaftor/ivacaftor use and require hypotensive therapy (Gramegna et al., 2022). Patients with CF associated with hypertension often require blood pressure control. Nisoldipine and nimodipine, which are dihydropyridine calcium channel blockers, are commonly utilized in clinical settings to regulate blood pressure. Previous studies have indicated that dihydropyridine calcium channel blockers are metabolized by CYP3A4 (Katoh et al., 2000). Nisoldipine, a cardiovascular medication mainly used to manage hypertension, is primarily metabolized in humans by CYP3A4 (Yuan et al., 2014). Nimodipine, also a cardiovascular drug, is mainly used to dilate cerebral blood vessels and has an inhibitory effect on CYP3A4 (Kong et al., 2023; Tomassoni et al., 2009). Ivacaftor undergoes metabolism by the CYP3A enzyme family, primarily converting to the active metabolite M1 (Garg et al., 2019). The aim of our study was to utilize an UPLC-MS/MS assay that was able to accurately detect the concentrations of ivacaftor and M1 to explore the effects of various drugs that may undergo DDI on the metabolism of ivacaftor *in vitro* and *in vivo*. On this basis, we also explored the mechanism of inhibition of ivacaftor by nisoldipine and nimodipine.

First, we selected 79 drugs with potential inhibitory activity as inhibitors in an established RLM culture system. These drugs may affect the metabolism of ivacaftor, so we performed experiments to clarify the inhibitory capacity of these drugs. Our *in vitro* findings indicated that 20 drugs inhibited ivacaftor metabolism by at least 80%. These drugs may have more substantial impacts on ivacaftor metabolism. Of the 20 drugs in question, 6 were identified as dihydropyridine calcium channel blockers, which are cardiovascular drugs utilized to regulate blood pressure. Further studies were conducted to investigate the inhibitory effect of these 6 dihydropyridine calcium channel blockers on the metabolism of ivacaftor. As illustrated in Figure 4, the IC_50_ curves and values of the 6 dihydropyridine calcium channel blockers on ivacaftor had been obtained through *in vitro* studies in RLM. The findings indicated that 4 substances with IC_50_ values < 10 μM had moderate inhibitory effects on ivacaftor in RLM: nicardipine (1.02 μM), lacidipine (2.17 μM), nimodipine (4.57 μM), and nisoldipine (6.55 μM). Considering the widespread clinical use of nisoldipine and nimodipine and the fact that there were reports in the literature of DDI of these drugs with cyclosporine and statins (Kong et al., 2023; Zhou et al., 2014), we further scrutinized the inhibition mechanism of nisoldipine and nimodipine against ivacaftor using RLM and HLM. The Lineweaver-Burk plots depicted in Figure 6 indicated that the mechanism of nisoldipine on ivacaftor in RLM and HLM worked through a mixed type of inhibition mechanism, including non-competitive and competitive inhibition. However, Figure 7 showed that the mechanism of inhibition of nimodipine on ivacaftor in RLM and HLM worked through competitive inhibition. Although both drugs are dihydropyridine calcium channel blockers and both affect L-type voltage-gated calcium channels, it is possible that differences in the structure and interaction binding sites of the drugs may be responsible for the different mechanisms of inhibition (Calcium Channel Blockers, 2012).

This *in vitro* study provided a basis for evaluating the pharmacokinetics of ivacaftor with or without nisoldipine or nimodipine in rats. Subsequently, we performed *in vivo* experiments in rats using nisoldipine and nimodipine as inhibitors of ivacaftor. One study reported an AUC_(0–24h)_ of 22,177 ng/mL*h for ivacaftor in male rats following gavage of 10 mg/kg ivacaftor alone (Harbeson et al., 2017). In our *in vivo* experiments, 10 mg/kg of ivacaftor was administered by gavage to rats and the results showed significant differences in pharmacokinetic parameters between group B and C and control group respectively. As shown in Table 3, the AUC_(0-t)_ of ivacaftor alone was 8,408.56 ng/mL*h, which was significantly lower than that of the above mentioned. This may be due to the fact that we used CMC-Na as a solvent, whereas this study used PEG400 as a solvent (Harbeson et al., 2017). The difference in solvent may be the reason for this discrepancy. In a more recent study, the researchers used aqueous suspension as the solvent, and after comparing the AUC_(0–24)_ normalized to a dose of 1 mg/kg, there was less of a difference than in our study (Pozniak et al., 2024). When ivacaftor was administered in combination with nisoldipine or nimodipine, CL_z/F_ was significantly decreased in rats, while AUC_(0-t)_ was significantly increased. However, there was no statistically significant change in t_1/2_ for ivacaftor. This may be attributed to the inhibitory effects of nimodipine and nisoldipine on P-glycoprotein (Zhang et al., 2003; Bailey and Dresser, 2004), which affects ivacaftor efflux. Moreover, our results showed the significant inhibition of ivacaftor metabolism by nimodipine and nisoldipine, which may be due to the important role of CYP450 enzymes. The extant evidences indicate that nimodipine and nisoldipine are metabolized via CYP3A4, so the competitive inhibition of CYP3A4 may underlie the augmented plasma exposure (Yuan et al., 2014; Kong et al., 2023). In addition, nisoldipine and nimodipine reduced MR values, while pharmacokinetic parameters of M1 were not significantly different between these groups.

The results of our study demonstrated that both nisoldipine and nimodipine exhibited potent inhibition with an IC_50_ value of less than 10 μM in both RLM and HLM. The inhibition mechanism of nisoldipine in both RLM and HLM was a mixed type of non-competitive and competitive inhibition, whereas nimodipine was competitively inhibition in both. Although both drugs produced consistent inhibitory effects on ivacaftor metabolism, however, there were limitations in our experimental results considering the interspecies differences between rats and humans, such as differences in P450 enzyme composition and protein binding. Therefore, further studies are needed in the future to clarify the effects of these drugs on the metabolism of ivacaftor in humans. In addition, the results of *in vitro* experiments showed that nisoldipine and nimodipine had significant inhibitory effects on ivacaftor metabolism, but the results of pharmacokinetic experiments showed that the t_1/2_ of ivacaftor by these two drugs was less affected. Nisoldipine and nimodipine were the most potent inhibitors of ivacaftor metabolism among these 79 drugs, but they did not produce clinically relevant DDI when it was used in combination with these two drugs, and no dosage adjustment was required in the absence of any significant adverse effects. Our findings suggest that ivacaftor is less likely to cause DDI when used in combination with these drugs. Our study helps to promote rational clinical use of medications and improve the quality of life of CF patients.

## 5 Conclusion

79 drugs that may be used in combination with ivacaftor and affect metabolism were screened. The results of the *in vitro* study showed that nisoldipine and nimodipine strongly inhibited the metabolism of ivacaftor through mixed and competitive inhibitory mechanisms, respectively. Results of *in vivo* studies showed that the combined use of nisoldipine and nimodipine resulted in a clinically irrelevant DDI. The results of the study help clinicians rationalize the use of medications.

## Data Availability

The original contributions presented in the study are included in the article/Supplementary Material, further inquiries can be directed to the corresponding authors.
