# Corrigendum: Inhibitory effects of calcium channel blockers nisoldipine and nimodipine on ivacaftor metabolism and their underlying mechanism

**DOI:** 10.3389/fphar.2024.1495855

**Published:** 2024-09-27

**Authors:** Hailun Xia, Xinhao Xu, Jie Chen, Hualu Wu, Yuxin Shen, Xiaohai Chen, Ren-ai Xu, Wenzhi Wu

**Affiliations:** First Affiliated Hospital of Wenzhou Medical University, Wenzhou, Zhejiang, China

**Keywords:** cystic fibrosis, ivacaftor, nisoldipine, nimodipine, drug-drug interaction, pharmacokinetics

In the published article, there was an error in Figure 7 as published. During the mapping process, the wrong unit of concentration value was used. Previous mapping used mg/mL as the unit of concentration for the X-value, which is inconsistent with the unit used for the figure, and is now converted to μM. The resulting K_i_-values change from 0.17 and 0.30 to 3.26 and 5.87, respectively. The corrected Figure 7 and its caption appear below.

**FIGURE 7:**
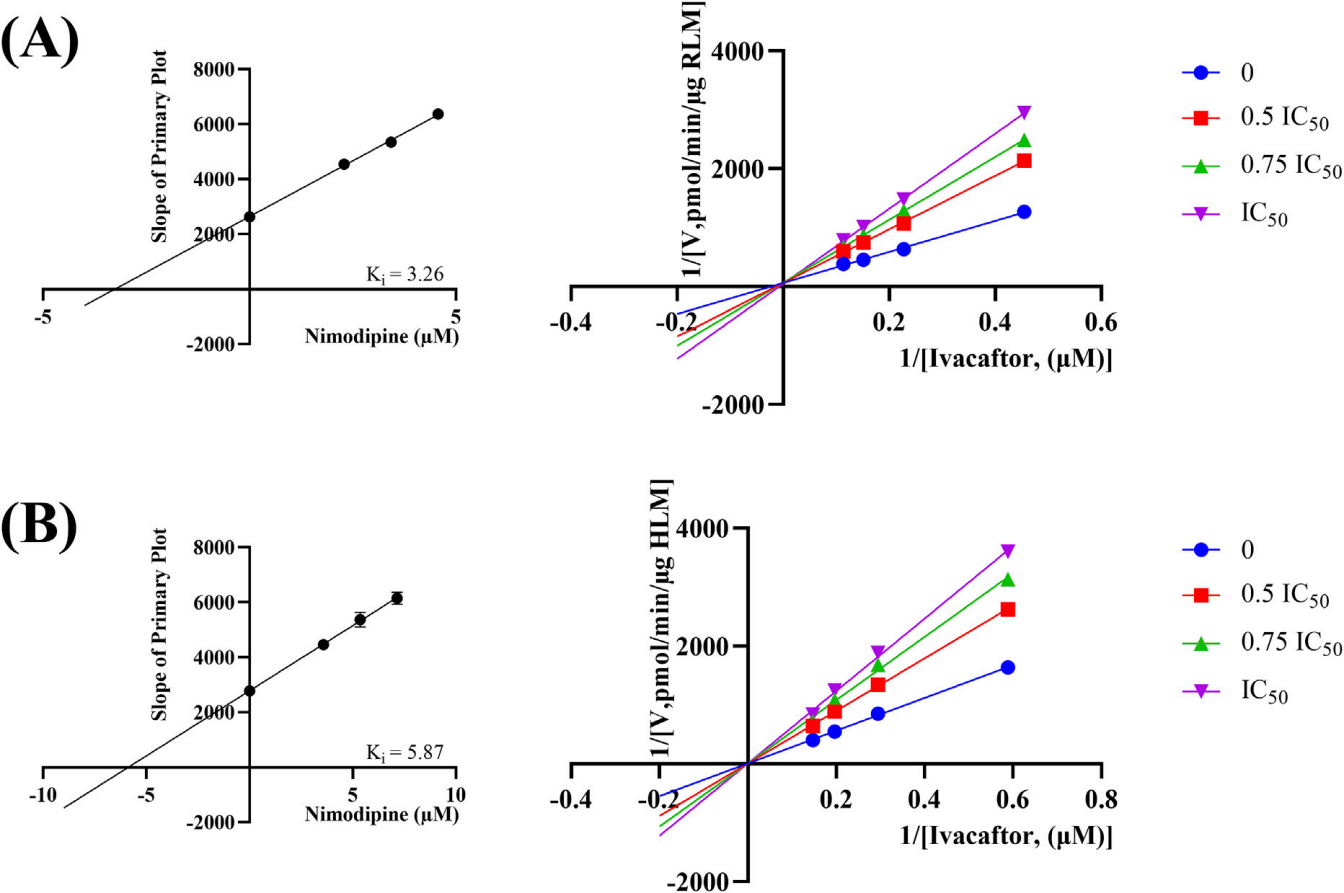
Lineweaver-burk plot, secondary diagram of K_i_ inhibiting ivacaftor metabolism at different concentrations of nimodipine in RLM **(A)** and in HLM **(B)**.

In the published article, there was an error in Table 2 as published. During the mapping process, the wrong unit of concentration value was used for Nimodipine. Previous mapping used mg/mL as the unit of concentration for the X-value, which is inconsistent with the unit used in the table, and is now converted to μM. The resulting K_i_-values change from 0.17 and 0.30 to 3.26 and 5.87, respectively. The corrected Table 2 and its caption appear below.

**TABLE 2:** The IC_50_ values and inhibitory effects of nisoldipine and nimodipine on ivacaftor metabolism in RLM and HLM.

Inhibitors		IC_50_ values (μM)	Inhibition type	K_i_ (μM)	αK_i_ (μM)	α
Nisoldipine	RLM	6.55	non-competitive inhibition and competitive inhibition	3.35	8.48	2.53
	HLM	9.10	non-competitive inhibition and competitive inhibition	3.92	35.40	9.03
Nimodipine	RLM	4.57	competitive inhibition	3.26		
	HLM	7.15	competitive inhibition	5.87		

In the published article, there was an error. During the mapping process, the wrong unit of concentration value was used. Previous mapping used mg/mL as the unit of concentration for the X-value and is now converted to μM. The resulting K_i_-values change from 0.17 and 0.30 to 3.26 and 5.87, respectively.

A correction has been made to **3 Results**, *3.3 Nisoldipine and nimodipine inhibited ivacaftor metabolism in RLM and in HLM through different inhibitory mechanisms*. This sentence previously stated:

“The K_i_ values were 0.17 and 0.30, respectively (Table 2).”

The corrected sentence appears below:

“The K_i_ values were 3.26 and 5.87, respectively (Table 2).”

The authors apologize for these errors and state that these do not change the scientific conclusions of the article in any way. The original article has been updated.

