# Supplementary material for: Inhibitory effects of calcium channel blockers nisoldipine and nimodipine on ivacaftor metabolism and their underlying mechanism
Source: Front Pharmacol. 2024 Sep 12;15:1403649. doi: 10.3389/fphar.2024.1403649 (PMC11424460; doi:10.3389/fphar.2024.1403649)
Supplement: Supplementary file 1 [file Table1.DOCX]

***Supplementary information***

**Inhibitory effects of calcium channel blockers nisoldipine and nimodipine on ivacaftor metabolism and their underlying mechanism**

Supplementary Table S1

**Supplementary Table S1.** The information about the 79 drugs.

| **Name** | **CAS** | **Metabolic rate**  **(Mean ± SD, % of control)** |
| --- | --- | --- |
| (-)-Epigallocatechin gallate | 989-51-5 | 72.21 ± 13.75 |
| 2-(4-Hydroxyphenyl) acetic acid | 156-38-7 | 101.32 ± 0.94 |
| Amiodarone | 1951-25-3 | 5.73 ± 0.85 |
| Andrographolide | 5508-58-7 | 81.58 ± 1.22 |
| Apigenin | 520-36-5 | 58.35 ± 2.14 |
| Apixaban | 503612-47-3 | 83.91 ± 3.91 |
| Artemether | 71963-77-4 | 41.88 ± 3.81 |
| Baicalein | 491-67-8 | 15.24 ± 0.00 |
| Baicalin | 21967-41-9 | 50.95 ± 0.08 |
| Berberine | 2086-83-1 | 109.72 ± 0.72 |
| Bergenin | 477-90-7 | 142.63 ± 33.46 |
| Betaine | 107-43-7 | 100.10 ± 4.97 |
| Bosentan | 147536-97-8 | 131.49 ± 2.21 |
| Caffeic acid | 331-39-5 | 106.41 ± 1.94 |
| cangrelor | 163706-06-7 | 105.13 ± 0.21 |
| Carvedilol | 72956-09-3 | 12.87 ± 0.57 |
| Chrysin | 480-40-0 | 74.50 ± 20.73 |
| Cilostazol | 73963-72-1 | 13.12 ± 0.36 |
| Cimetidine | 51481-61-9 | 62.59 ± 8.46 |
| Curcumin | 458-37-7 | 8.19 ± 0.34 |
| Dabigatran | 211914-51-1 | 104.42 ± 27.66 |
| Daidzein | 486-66-8 | 105.78 ± 2.90 |
| Daphnoretin | 2034-69-7 | 69.60 ± 0.85 |
| Dapoxetine | 119356-77-3 | 19.76 ± 0.64 |
| Dilthiazem | 42399-41-7 | 26.04 ± 1.00 |
| Dronedarone | 141626-36-0 | 4.76 ± 0.94 |
| Epicatechin | 490-46-0 | 100.31 ± 2.66 |
| Etodolac | 41340-25-4 | 98.19 ± 1.07 |
| Fangchinoline | 33889-68-8 | 29.41 ± 4.61 |
| Felodipine | 72509-76-3 | 13.47 ± 1.71 |
| Fisetin | 528-48-3 | 44.37 ± 1.28 |
| Fluvastatin | 93957-54-1 | 93.16 ± 0.36 |
| Gallic acid | 149-91-7 | 95.98 ± 6.77 |
| Genistein | 446-72-0 | 50.39 ± 12.01 |
| Glimepiride | 93479-97-1 | 36.97 ± 1.02 |
| Hesperetin | 520-33-2 | 31.12 ± 2.75 |
| Hesperiden | 520-26-3 | 104.17 ± 3.09 |
| Isorhamnetin | 480-19-3 | 65.68 ± 3.49 |
| Kaempferol | 520-18-3 | 66.02 ± 4.43 |
| Lacidipine | 103890-78-4 | 14.13 ± 1.07 |
| Lercanidipine | 100427-26-7 | 26.70 ± 7.18 |
| Limonin | 1180-71-8 | 19.90 ± 0.36 |
| Lornoxicam | 70374-39-9 | 79.39 ± 3.06 |
| Losartan | 114798-26-4 | 35.88 ± 1.19 |
| Lovastatin | 75330-75-5 | 17.75 ± 0.36 |
| Luteolin | 491-70-3 | 95.78 ± 7.92 |
| Lycopene | 502-65-8 | 7.16 ± 0.77 |
| Matrine | 519-02-8 | 80.23 ± 12.45 |
| mavacamten | 1642288-47-8 | 94.17 ± 0.21 |
| Mexiletine | 31828-71-4 | 95.32 ± 2.42 |
| Myricetin | 529-44-2 | 2.38 ± 0.15 |
| Naringenin | 480-41-1 | 42.57 ± 2.14 |
| Naringin | 10236-47-2 | 100.87 ± 17.42 |
| Nicardipine | 55985-32-5 | 5.28 ± 0.07 |
| Nifedipine | 21829-25-4 | 19.31 ± 0.28 |
| Nimodipine | 66085-59-4 | 7.84 ± 0.43 |
| Nisoldipine | 63675-72-9 | 7.29 ± 4.05 |
| Nitrendipine | 39562-70-4 | 27.00 ± 0.21 |
| PF-04971729 | 1210344-57-2 | 92.72 ± 3.41 |
| Piperine | 94-62-2 | 203.66 ± 9.93 |
| Prasugrel | 150322-43-3 | 31.93 ± 1.49 |
| Propafenone | 54063-53-5 | 54.95 ± 0.92 |
| Quercetin | 117-39-5 | 57.86 ± 5.58 |
| Quinidine | 56-54-2 | 37.44 ± 4.20 |
| Resveratrol | 501-36-0 | 18.69 ± 0.46 |
| Rivaroxaban | 366789-02-8 | 85.62 ± 0.92 |
| Rutin | 153-18-4 | 91.63 ± 0.31 |
| Selexipag | 475086-01-2 | 28.78 ± 2.21 |
| Shikonin | 517-89-5 | 41.65 ± 3.13 |
| Silibinin | 22888-70-6 | 54.73 ± 1.11 |
| Sitagliptin | 486460-32-6 | 97.11 ± 2.13 |
| Sophocarpine | 6483-15-4 | 93.46 ± 2.14 |
| Sophoridine | 6882-68-4 | 109.11 ± 5.90 |
| Telmisartan | 144701-48-4 | 14.98 ± 1.00 |
| Tetrandrine | 518-34-3 | 71.96 ± 16.27 |
| Verapamil | 52-53-9 | 26.85 ± 1.56 |
| Vericiguat | 1350653-20-1 | 76.32 ± 1.42 |
| Vortioxetine | 508233-74-7 | 3.17 ± 0.50 |
| Warfarin | 81-81-2 | 95.32 ± 3.41 |

Supplementary Table S2

**Supplementary Table S2.** Protein concentration of RLM and HLM used in the experiment

|  | Protein concentration |
| --- | --- |
| RLM | 35.25 mg/mL |
| HLM | 20.00 mg/mL |
